# Human olfactory mesenchymal stromal cell transplantation ameliorates experimental autoimmune encephalomyelitis revealing an inhibitory role for IL16 on myelination

**DOI:** 10.1186/s40478-022-01316-9

**Published:** 2022-01-29

**Authors:** Susan L. Lindsay, Aleksandra M. Molęda, Lindsay M. MacLellan, Siew Min Keh, Daniel E. McElroy, Christopher Linington, Carl S. Goodyear, Susan C. Barnett

**Affiliations:** 1 Institute of Infection, Inflammation and Immunity, University of Glasgow, Sir Graeme Davies Building, 120 University Place, Glasgow, G12 8TA UK; 2 Department of Otolaryngology, Elizabeth University Hospital Glasgow, Glasgow, G51 4TF Queen UK

**Keywords:** Human mesenchymal stromal cells, Olfactory mucosa, Multiple sclerosis, Experimental autoimmune encephalomyelitis, Myelination, Interleukin 16

## Abstract

One of the therapeutic approaches for the treatment of the autoimmune demyelinating disease, multiple sclerosis (MS) is bone marrow mesenchymal stromal cell (hBM-MSCs) transplantation. However, given their capacity to enhance myelination in vitro, we hypothesised that human olfactory mucosa-derived MSCs (hOM-MSCs) may possess additional properties suitable for CNS repair. Herein, we have examined the efficacy of hOM-MSCs versus hBM-MSCs using the experimental autoimmune encephalomyelitis (EAE) model. Both MSC types ameliorated disease, if delivered during the initial onset of symptomatic disease. Yet, only hOM-MSCs improved disease outcome if administered during established disease when animals had severe neurological deficits. Histological analysis of spinal cord lesions revealed hOM-MSC transplantation reduced blood–brain barrier disruption and inflammatory cell recruitment and enhanced axonal survival. At early time points post-hOM-MSC treatment, animals had reduced levels of circulating IL-16, which was reflected in both the ability of immune cells to secrete IL-16 and the level of IL-16 in spinal cord inflammatory lesions. Further in vitro investigation revealed an inhibitory role for IL-16 on oligodendrocyte differentiation and myelination. Moreover, the availability of bioactive IL-16 after demyelination was reduced in the presence of hOM-MSCs. Combined, our data suggests that human hOM-MSCs may have therapeutic benefit in the treatment of MS via an IL-16-mediated pathway, especially if administered during active demyelination and inflammation.

## Introduction

Multiple sclerosis (MS) is a chronic disease in which repeated episodes of inflammation in the central nervous system (CNS) result in widespread demyelination associated with varying degrees of irreversible axonal injury and loss [1, 2]. Current treatments target the inflammatory element of MS, a strategy that is beneficial for patients with early relapsing–remitting MS but is largely ineffective in progressive forms of the disease [1, 3, 4]. Demyelination increases axonal susceptibility to damage by inflammatory mediators and treatment strategies designed to suppress disease activity, which allow or promote endogenous remyelination are considered a rational approach to enhance axonal function and survival [2, 5].

Several studies discuss pharmacological approaches to enhance remyelination by stimulating oligodendrocyte progenitor cell (OPC) proliferation, differentiation, and survival in the CNS however, mesenchymal stromal cell (MSC) transplantation provides an alternative strategy. There are several reports demonstrating the beneficial effects of syngeneic [4, 6] or human [7, 8] MSC transplantation in experimental autoimmune encephalomyelitis (EAE), an animal model of MS. These include reduced inflammation, demyelination, axonal loss, as well as corresponding increases in remyelination. These beneficial effects are generally attributed to immunomodulation in the periphery, although MSCs may also be recruited into the CNS to secrete soluble neuroprotective factors that support endogenous tissue repair and remyelination [1, 2, 4]. Indeed, MSCs modulate the local tissue microenvironment, via their release of neuroprotective and pro-oligodendrogenic factors and can inhibit infiltrating pathogenic immune responses [9]. Furthermore, promising results have been seen in human trials of autologous transplantation of MSCs in secondary progressive patients, illustrating their safety [10, 11]. There has also been evidence of neuroprotection from structural and functional improvements, which strongly supports their future use as a treatment [10, 12, 13]. These pre-clinical studies provide a functional basis for the beneficial effects of autologous MSC transplantation already reported in MS patients and support its future use as a routine intervention.

We have identified another population of MSCs from human biopsies of olfactory mucosa (OM), termed hOM-MSCs [14–16]. The olfactory system is well known to have inherent reparative properties since it is capable of supporting neurogenesis throughout life, attributed to both endogenous stem cell populations and a specialised glial cell type [17]. Human OM cell/tissue transplantation has already been shown to be safe in phase 1 clinical trials for the treatment of spinal cord injured patients [18, 19]. Human OM-MSCs have been shown to have similar antigenic and differentiation properties to those of classical human BM-MSCs (hBM-MSCs) [14, 15, 20, 21]. However, unlike hBM-MSCs, they can be grown in large numbers easily and have been shown to enhance myelination of dissociated rat embryonic spinal cord cell cultures [14]. Notably, in an animal model of spinal cord injury (SCI) hOM-MSCs enhanced Schwann cell remyelination of spared tissue fibres, lending to a faster recovery of animal co-ordinated stepping [22]. Our prior data also showed that hOM-MSCs secrete specific anti-inflammatory chemokines that skew microglia to an anti-inflammatory phenotype [14, 15].

This in vitro data has therefore demonstrated that human hOM-MSCs may be an alternative MSC candidate for transplant-mediated repair in clinical trials. Herein, we have compared the therapeutic benefit of hOM-MSCs to hBM-MSCs in the amelioration of EAE. Although both hOM- and hBM-MSCs improved animal outcome if administered during mild EAE, only hOM-MSCs proved beneficial when delivered during severe disease. Moreover, animals treated with hOM-MSCs had reduced levels of inflammatory cellular infiltrates within spinal cord lesions accompanied with less disruption of the blood–brain barrier at early time points post-treatment. Animals which received hOM-MSCs had reduced levels of circulating IL-16, which corresponded to a reduced ability of immune cells to secrete IL-16 and the extent of IL-16 present in the spinal cord inflammatory lesions. Notably, IL-16 prevented oligodendrocyte differentiation and myelination in vitro, suggesting that this axis may be important in the hOM-MSC pro-repair mechanism-of-action. This work supports the use of hOM-MSCs as a novel candidate for clinical translation for the treatment of MS.

## Material and methods

### EAE induction

A total of eighty-five female C57Bl/6 J mice were purchased from Harlan Laboratories (Loughborough, UK). All mice were housed under a 12-h light/dark cycle with ad libitum access to food and water in pathogen-free conditions. All experimental procedures were performed in accordance with the UK Animals (Scientific Procedures) Act 1986. All applicable international, national, and/or institutional guidelines for the care and use of animals were followed. The research protocol was approved by the Ethical Committee for Animal Experimentation in the University of Glasgow, UK.

EAE was induced in female mice (7–8 weeks of age, weighing 18.5 ± 1.5 g) by subcutaneous injection at one site at the tail base with an emulsion (100 µl total) containing 200 µg recombinant rat myelin oligodendrocyte glycoprotein protein spanning amino acids 1–125 (MOG1–125) in complete Freund’s adjuvant (Sigma-Aldrich) supplemented with 200 µg Mycobacterium tuberculosis (strain H37RA; Difco). Mice were injected intraperitoneally with 200 ng pertussis toxin (Enzo) in 100 µl of phosphate buffer saline solution (PBS, pH 7.6) immediately, and 48 h after the immunisation. The mice were scored daily for clinical manifestations of EAE on a half point scale of 0–5 [23, 24]. hOM-MSCs or hBM-MSCs (1 × 10^6^ cells/100 μl) or PBS (100 μl) were injected at an early time point following disease onset, only when animals showed signs of clinical disease (score of 1; loss of tail tone) or alternatively, at a later time point in the EAE clinical course when animals had more severe neurological deficits (average score of 2.5; hind limb paralysis). This treatment strategy prevented the inclusion of asymptomatic animals.

### Human tissue biopsies

All procedures performed involving human participants were in accordance with the ethical standards of the University of Glasgow and the 1964 Helsinki Declaration and its later amendments or comparable ethical standards. Human olfactory tissue biopsies or bone marrow aspirate were obtained with South Glasgow and Clyde Research Ethics Committee and Central Office for Research Ethics approval (07/S0710/24) and informed patient consent from both males and females. Olfactory tissue biopsies were taken from patients undergoing nasal septoplasty/polypectomy surgery (average age 46.5 years). Biopsies were taken from superior regions known to contain olfactory mucosa [25]. Biopsies were collected, purified, and grown as previously described [14]. After purification, (termed hOM‐MSCs) cells to be used in biodistribution studies were lentivirally infected using a MOI of 10 (Amsbio UK, LVP001) to produce GFP expressing cells. Lentiviral GFP infection was > 98% in all preparations [22]. Human BM aspirates were obtained from iliac crests of patients undergoing hip replacement (average age 56.3 years, which was not significantly different from donor patients of hOM-MSCs). BM was collected, purified, and  hBM-MSCs grown as previously described [15, 20].

### Immunohistochemistry

Mice under deep anaesthesia were transcardially perfused with 4% paraformaldehyde (PFA). Spinal cords were removed and post-fixed by immersion in the same fixative containing 30% sucrose at 4 °C for 24 h, then washed and left in PBS containing 30% sucrose at 4 °C until being frozen. Five mm spinal cord tissue blocks encompassing regions L2 to S1 were frozen on dry ice in OCT and consecutive 5 μm thick sections were cut and numbered in order of cutting. Inflammatory demyelination was assessed by staining for myelin basic protein (MBP) with anti-MBP antibody (1:200, BioRad) and axonal loss was assessed using anti-SMI-31 (1:1000, Bio-Legend). Inflammatory infiltrates were analysed using anti–mouse CD45 (1:100, R&D systems), anti–mouse CD4 (R&D systems) and anti–mouse CD11b (1:500, Abcam) antibodies, which identify T cells and microglia/macrophages or haematoxylin and eosin staining, following standard methods. Anti-glial fibrillary acidic protein (GFAP, 1:500, Dako) was used for the detection of astrocytes. Anti-laminin (α1 subunit) (1:500, Sigma) was used for the detection of the blood–brain barrier and is known to be upregulated during EAE [26]. IL-16 was detected using anti-rat/mouse IL-16 (1:200, Caltag-Medsystems). All primary antibodies were detected with appropriate conjugated secondary antibody (1:500, Thermofisher) according to established standard protocols. Sections were mounted using VECTASHIELD antifade medium containing DAPI (Vector labs).

### Quantification of spinal cord sections

Quantification of antibody labelling was performed using sequential sections across the same spinal cord regions in all experimental groups. Two images per section, with a minimum of three sections per animal were quantified. Images were captured at 20 × magnification with an Olympus BX51 microscope using Occular software and analysed using Fiji (Image J). The captured images were coded and quantified in a blinded manner. For thresholding analysis, the white matter region of interest (ROI) in each image was manually outlined using ImageJ and outside regions cleared. Binary thresholding measurements were made of both the total ROI and of any positive staining and converted into the number of black pixels. Data was expressed as % of positive staining per field of view within the total number of positive elements exclusively in the white matter ROI. For semi-quantification of EAE spinal cords, sections stained with MBP/SMI/DAPI were scored according to the level of disease disruption (score 0 = healthy tissue, few DAPI nuclei in white matter, normal SMI/MBP staining; score 1 = mild disruption: some DAPI nuclei in white matter regions although little disruption to SMI/MBP; score 2 = moderate disruption: increased amount of DAPI nuclei in obvious inflammatory lesions, loss of SMI/MBP staining; score 3 = severe disruption: large number of DAPI nuclei in lesions extending into a large area of the white matter. Clear disruption of SMI/MBP). Scores were averaged for each animal. For quantification of SMI or MBP loss, using Image J, a grid containing 130 crosses was applied across each white matter ROI. Each cross falling on a region of abnormal pattern of staining was counted, and the number of regions divided by the total number of crosses covering the entire ROI and expressed as percentage of abnormal staining/tissue area per field of view. Quantification of H&E stained inflammatory infiltration was made by counting the number of nuclei present within the same spinal cord white matter ROI in at least 3 spinal cord sections/animal, imaged on a Nikon ECLIPSE E200 microscope (Nikon Instrument) equipped with a DS-2Mv camera at 40× magnification.

### BBB disruption measured by FITC-dextran labelling

Severe EAE mice were injected with a solution containing 71-kDa FITC-labelled dextran (5 mg/100 μl, Sigma-Aldrich) into the tail vein. Fifteen minutes later, the mice under deep anaesthesia were perfused as previously described and the spinal cords were collected immediately. The cords were processed by post-fixation as described above, and a 5 mm block encompassing the T2 to L2 segmental level was used for analysis. Sixty-µm-sagittal cryostat sections were cut and analysed categorically by light microscopy (Leica Microsystems) with 20 × lens. Sections were scored as follows: 0 = no traces of FITC-dextran disruption, dextran located within blood vessels; 1 = traces of light disruption, however numerous blood vessels still show accumulation of labelling; 2 = marked dextran disruption throughout the white matter; 3 = extensive disruption with FITC labelling throughout the white matter of the cord and no presence of blood vessel labelling. Each animals’ average disruption severity score was calculated by summing the assigned numerical value of each section and dividing it by the total number of sections per animal.

### In vitro de/myelinating assays

Myelinating rat embryonic CNS cultures were grown using methods previously described [14, 27]. Briefly, on day 12 cultures were treated with 100 ng/ml IL-16 (#200-16A, Peprotech) every other day until day 28. Cultures were then fixed and stained and quantified for levels of myelination. Quantification was carried out using CellProfiler Image Analysis software (Broad Institute) [28]. For neurite density, the threshold level pixel value for SMI31 immunoreactivity (IR) was divided by the total number of pixels. The percentage of myelinated axons (PLP) was measured using CellProfiler, which uses pattern recognition software to distinguish between linear myelinated internodes and oligodendrocyte cell bodies. In this manner, we track the co‐expression of myelin sheaths (PLP) and axons (SMI31) and calculate this percentage of myelinated fibers. All experiments were carried out at least three times in duplicate. All CellProfiler pipelines are available at https://github.com/muecs/cp.

For demyelinating assays (DeMy), on day 24 cultures were treated with anti-MOG (Z2 hybridoma, IgG2a [29]) and 100 μg/ml rabbit serum complement (Millipore). The following day, the culture supernatant was removed and replaced with fresh DMEM (4,500 mg/ml glucose) containing, 10 ng/ml biotin, 0.5% hormone mixture (1 mg/mL apo-transferrin, 20 mM putrescine, 4 μM progesterone, 6 μM selenium (formulation based on N2 mix of [30] 50 nM hydrocortisone and 10 μg/ml insulin (DM; all reagents from either Sigma or Life technologies, Paisley). After demyelination, cultures were treated twice either with DM or DM supplemented with conditioned media (CM) derived from hOM-MSCs or hBM-MSCs (harvested as previously described [14]), or non-demyelinated cultures were left as controls. Cultures were maintained for a further 5 days before being lysed for Western blot analysis.

### Culture of rat microglia and oligodendrocyte precursor cells (OPCs)

Sprague Dawley cortices were digested and grown in DMEM containing 10% FBS with 4.5 g/l glucose, L-glutamine, pyruvate, and 1% penicillin/streptomycin (DMEM-10%) using standard methods [31]. After 7–10 days, microglia and OPCs were purified by differential attachment [32]. Microglia were plated in DMEM-10% on poly-L-lysine (PLL, Sigma, 13 µg/ml) coated glass coverslips (VWR, 10 µg/ml) at 5 × 10^4^ cells/coverslip. Microglia were stained using anti-mouse CD4 (1:100, ThermoFisher Scientific) and anti-rabbit Iba-1 (1:100, Wako) using standard immunohistochemistry methods described above. OPCs were plated onto 13 mm PLL coated glass coverslips at 4 × 10^4^ cells/coverslip in DMEM-BS containing FGF2 (50 ng/mL) and PDGF (50 ng/mL) for 5 days, then treated in duplicate with IL-16 (100 ng/ml) or DMEM-BS for 4 days. On day 5, cells were immunolabelled with anti-rabbit NG2 (1:100, Abcam), anti O4 (1:100, IgM, hybridoma) or anti-rat PLP (1:100, AA3, hybridoma) and 10 images taken per coverslip with 2 coverslips per treatment, with an average of 200–300 cells/coverslips analysed. OPCs were stained using CD4 described above and co-stained using anti-O4 (1:100, IgM, hybridoma).

### Western blot

Supernatants were collected from confluent flasks containing similar number of MSCs following standard methods [14] or cells lysed using CelLytic M (Sigma) containing protease inhibitor cocktail (Sigma) and protein concentration determined (NanoDrop; Thermo Scientific). Samples were run on tris–acetate gels and transferred (iBlot©; Invitrogen). Membranes were blocked in 5% BSA and 0.2% Triton-X100 in TBS for 1 h before being incubated overnight at 4 °C with rabbit IL-16 (1:500, Caltag Medsystems) or mouse Caspase-3 (1:1000, ThermoFisher Scientific). Total protein was assessed using mouse β-actin (1:1000, Sigma) in IL-16 experiments or Ponceau S staining to quantify secreted protein level after transfer for Caspase-3. Band intensities were quantified using Image J and normalised to loading.

### Isolation of lymphocytes for Meso scale discovery assay

Inguinal lymph node cells were harvested from severe EAE animals treated with hOM-MSCs or hBM-MSCs or PBS either 5 days or 24 days post-injection. Single-cell suspensions were prepared by enzymatic digestion using 1 mg/ml collagenase D (Roche, UK) in Hanks’ balanced salt solution without calcium and magnesium. Cells were cultured in triplicate in 96-well round-bottomed plates at 3 × 10^5^ cells per well in complete Dulbecco's modified Eagle's medium. Cells were restimulated with medium as a control or 30 μg/ml MOG protein (1–125) for 24 h and supernatants collected and assayed using a 35 U-PLEX mouse biomarker group 1 array (#K15083K-1, Meso Scale Discovery, UK) following manufacturers guidelines. The kit allowed us to assay an extensive combination of mouse cytokines and chemokines that are involved in inflammatory biological processes including the Th1, Th2, and Th17 pathways. The 35 cytokines analysed were: EPO, GM-CSF, IFN-γ, IL-1β, IL-2, IL-4, IL-5, IL-6, IL-9, IL-10, IL-12/IL-23p40, IL-12p70, IL-13, IL-15, IL-16, IL-17A, IL-17A/F, IL-17C, IL-17E/IL-25, IL-17F, IL-21, IL-22, IL-23, IL-27p28/IL-30, IL-31, IL-33, IP-10, KC/GRO, MCP-1, MIP-1α, MIP-1β, MIP-2, MIP-3α, TNF-α, VEGF-A.

### IL-16 ELISA

Serum samples were collected from severe EAE animals which had been treated with hOM-MSCs or hBM-MSCs or PBS either 5 days or 24 days post-injection. Levels of IL-16 were assayed using Mouse IL-16 ELISA (R&D systems, UK) following manufacturers guidelines.

### Statistical analysis

Parametric data are presented as means ± SEM, non-parametric as box and whisker plots. Differences between groups were statistically tested using the software package GraphPad Prism 6 (GraphPad Software Inc., San Diego, CA, USA). The applied statistical procedures are provided in the figure legends. In EAE experiments, data derived from each animal is represented by individual data points on the graph. In in vitro experiments, primary cultures set up from individual animal batches were considered an n number and each experiment was carried out between 3–5 times. Sample size calculations for EAE experiments were performed using a conventional protocol as previously detailed [33]. This calculation assumed that there were no differences in standard deviations between groups and that detection of 20% change is derived with 80% power at a 5% level of significance, for two‐sided significance tests. EAE animal numbers were set up accordingly to ensure that for each treatment the group size from which data was obtained was appropriate. p values < 0.05 were considered statistically significant. The following symbols are used to indicate the level of significance: *p < 0.05, **p < 0.01, ***p < 0.001.

## Results

### hOM-MSCs ameliorate progression of severe EAE

**Fig. 1:**
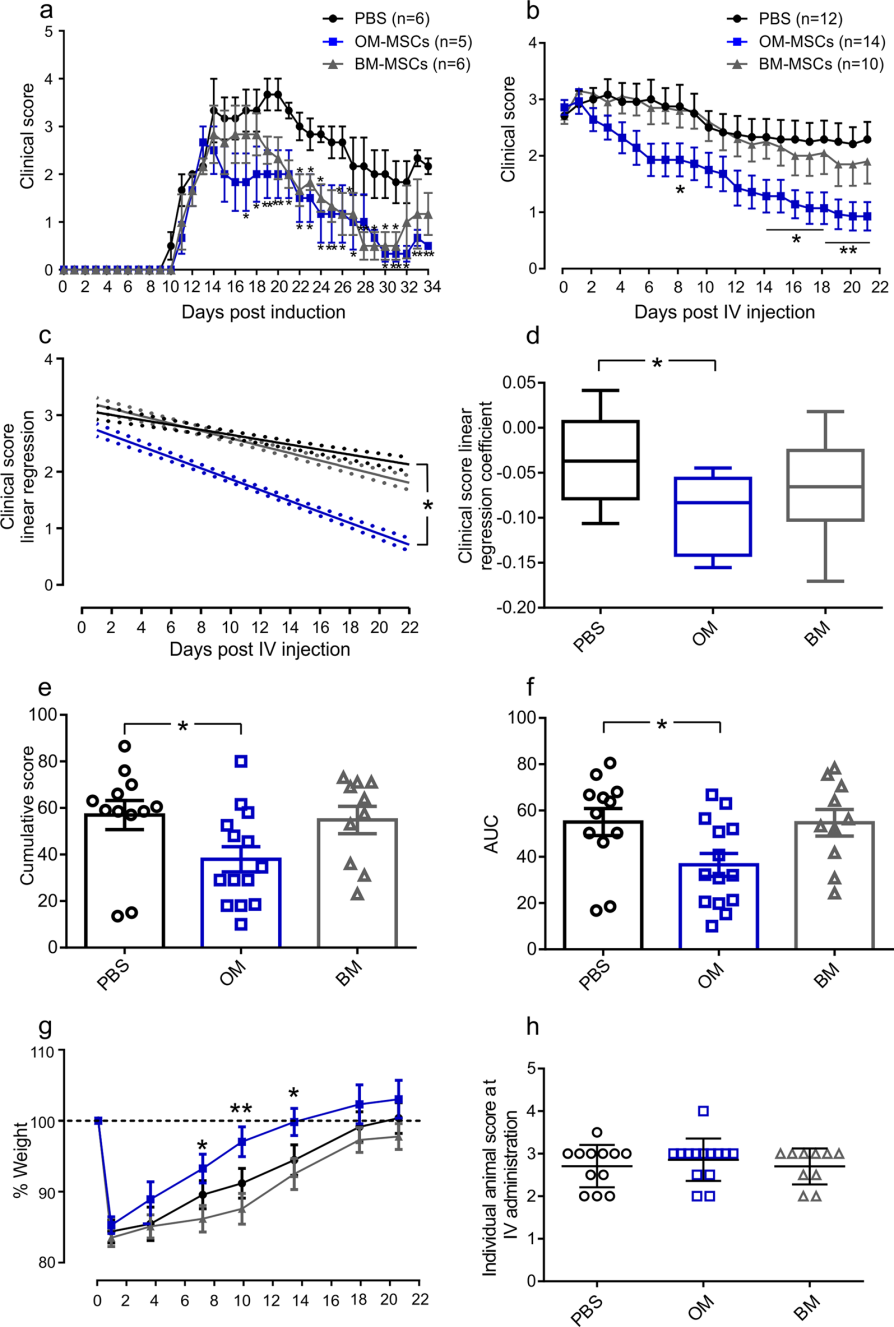
hOM-MSCs ameliorate EAE better than hBM-MSCs when administered during severe disease. **a** EAE clinical course after i.v. injection of 1 × 10^6^ hOM-MSCs (*n* = 5) or 1 × 10^6^ hBM-MSCs (*n* = 6) or PBS (*n* = 6) during early disease. Both hOM- and hBM-MSCs ameliorated EAE to a similar extent. **b** EAE clinical course after i.v. injection of 1 × 10^6^ hOM-MSCs (*n* = 14) or 1 × 10^6^ hBM-MSCs (*n* = 10) or PBS (*n* = 12) during severe disease. hOM-MSCs significantly ameliorated disease whilst hBM-MSCs did not (*p < 0.05, 2-way ANOVA with Tukey’s multiple comparison). **c** Individual linear regression lines fitted through severe disease animal EAE curves showed hOM-MSC treated animals have significantly steeper slopes compared to PBS animals. **d** Comparison of the coefficients confirmed significantly faster improvements in hOM-MSC injected animals compared with control, hBM-MSC injected animals had no significant difference (*p < 0.05, Kruskal–Wallis, Dunn’s multiple comparison test). **e** hOM-MSC treated animals had a significant reduction in cumulative disease scores and **f** the area under the curve (AUC) compared to PBS controls (*p < 0.05, ANOVA Tukey’s multiple comparison). **g** hOM-MSC injected animals had a faster recovery of pre-disease weight compared to PBS controls (*p < 0.05, **p < 0.01 2-way ANOVA with Tukey’s multiple comparison). **h** There were no significant differences in the distribution of animal score between the treatment groups

We compared the ability of hOM-MSCs and hBM-MSCs to ameliorate disease progression in EAE, monitoring clinical signs of disease on a daily basis for 24 days after systemic (i.v.) injection of hOM-MSCs or hBM-MSCs (1 × 10^6^ cells/100 μl) or PBS (100 μl). MSCs were injected early following disease onset, only when mice showed signs of clinical disease (score of 1; loss of tail tone). There were no significant differences between the day of onset between groups. MSCs were alternatively injected at a later time point when animals had developed at least partial hind limb paralysis (average score of 2.5). hOM- and hBM-MSCs ameliorated EAE to a similar extent compared to PBS injected controls when administered early (Fig. 1a), but only hOM-MSCs significantly improved recovery when cells were injected into animals with severe disease (Fig. 1b). Individual linear regression analysis of disease scores revealed hOM-MSC treated animals consistently have steeper slopes compared to PBS injected animals, corresponding to their faster recovery (Fig. 1c). In contrast, mice that received hBM-MSCs were not significantly different from PBS injected controls. Individual linear regression coefficients were calculated using the slopes of the regression lines for each animal (Fig. 1d). Comparison of the coefficients confirmed a significantly faster improvement in hOM-MSC injected animals compared with control, whereas hBM-MSC injected animals had no significant difference (Fig. 1d). Further analysis of animal EAE curve data showed a significant reduction in the area under the curve (AUC) and reduced cumulative disease scores of hOM-MSC injected animals compared to PBS control injected (Fig. 1e, f). In addition, animals which received hOM-MSCs had a faster improvement in weight compared to both PBS and hBM-MSC treated animals which correlated with improved animal health and scores (Fig. 1g). There were no significant differences between the disease scores of each experimental group at day of i.v. administration (Fig. 1h). These data show that only mice receiving hOM-MSCs produced a positive therapeutic benefit if administered when animals have active demyelination and severe neurological deficits.

### Treatment with hOM-MSCs reduce inflammation and axonal damage in the CNS during EAE

**Fig. 2:**
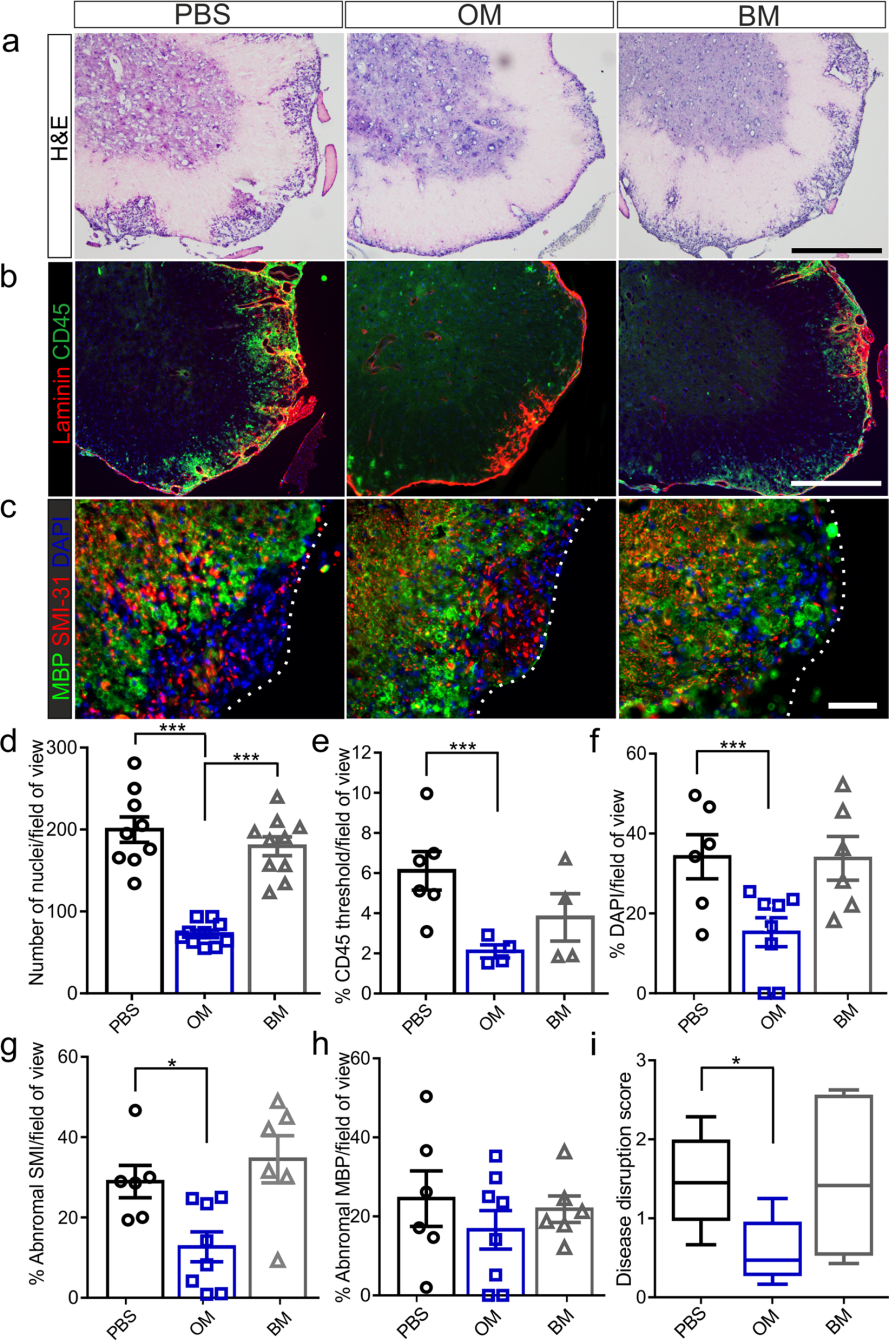
Treatment with hOM-MSCs reduces inflammation and axonal pathology in the spinal cord during severe EAE. Staining of endpoint lumbar spinal cord revealed that PBS- and hBM-MSCs treated animals had severe inflammation compared to the hOM-MSC treated group as shown by **a** H&E staining of inflammatory infiltrate (PBS, *n* = 9; hOM-MSCs, *n* = 10; hBM-MSCs, *n* = 10). **b** CD45 staining of infiltrating lymphocytes (shown in green) and Laminin (shown in red) (PBS, *n* = 6; hOM-MSCs, *n* = 4; hBM-MSCs, *n* = 4) or c. DAPI staining (shown in blue) (PBS, *n* = 6; hOM-MSCs, *n* = 8; hBM-MSCs, *n* = 6). Quantitative analysis showed that hOM-MSC injected animals had significantly fewer inflammatory cell regions as assessed by H&E (**d**) or CD45 (**e**) or DAPI (**f**) compared to PBS control animals. **c** Lumbar spinal cord tissue was stained for myelin (MBP, shown in green), axons (SMI-31, shown in red) and cellular infiltrate (DAPI, shown in blue) (PBS, *n* = 6; hOM-MSCs, *n* = 8; hBM-MSCs, *n* = 6). **g** Measurements of abnormal axonal pathology were significantly less in animals injected with hOM-MSCs compared to PBS animals. Animals transplanted with hBM-MSCs showed similar levels of axonal pathology as PBS control animals. **h** There were no significant differences in myelin pathology across the groups. *p < 0.05, **p < 0.01, ***p < 0.001, ANOVA, Tukey’s multiple comparison. **i** Spinal cord sections stained with SMI/MBP/DAPI were assigned a score according to the level of cellular infiltration and disruption. Animals injected with hOM-MSCs had significantly lower disease disruption scores compared to PBS control animals. Kruskall-Wallis with Dunn’s multiple comparison. Scale bars represent 100 μm (**a**, **b**) and 50 μm (**c**)

Analysis of experimental endpoint lumbar spinal cord tissue from animals treated during severe disease, revealed that PBS- or hBM-MSCs injected animals had more severe inflammation and axonal pathology compared to the hOM-MSC-injected group (Fig. 2). hOM-MSC-injected animals had fewer inflammatory foci (Fig. 2a) and infiltrating cells (Fig. 2d) compared to both PBS and hBM-MSC injected animals. Inflammatory infiltration was also significantly reduced in hOM-MSC injected animals compared to PBS injected controls when assessed using CD45, a pan lymphocyte marker (Fig. 2b, e). Quantification of DAPI nuclei within inflammatory lesions was similarly significantly reduced in hOM-MSC injected animals compared to PBS control animals corresponding with the reduced number of inflammatory cells as shown by H&E and CD45 quantification (Fig. 2c, f). Measurements of abnormal axonal pathology were significantly less in animals injected with hOM-MSCs compared to PBS controls (Fig. 2c, g), however there was no significant difference in myelin pathology (Fig. 2c, h). Animals transplanted with hBM-MSCs showed similar levels of axonal pathology as PBS injected control animals. Semi-quantification of histological sections revealed hOM-MSC transplanted animals had overall lower levels of disease severity compared to PBS control animals (Fig. 2i). These data suggest that in animals treated with hOM-MSCs there was a reduction in the level of inflammatory cell infiltration into the lumbar spinal cord and a prevention of axonal loss, correlating with a lower disease score at the endpoint of the experiment.

### Localisation of GFP-labelled hOM-MSCs within lumbar spinal cord and brain cortex

**Fig. 3:**
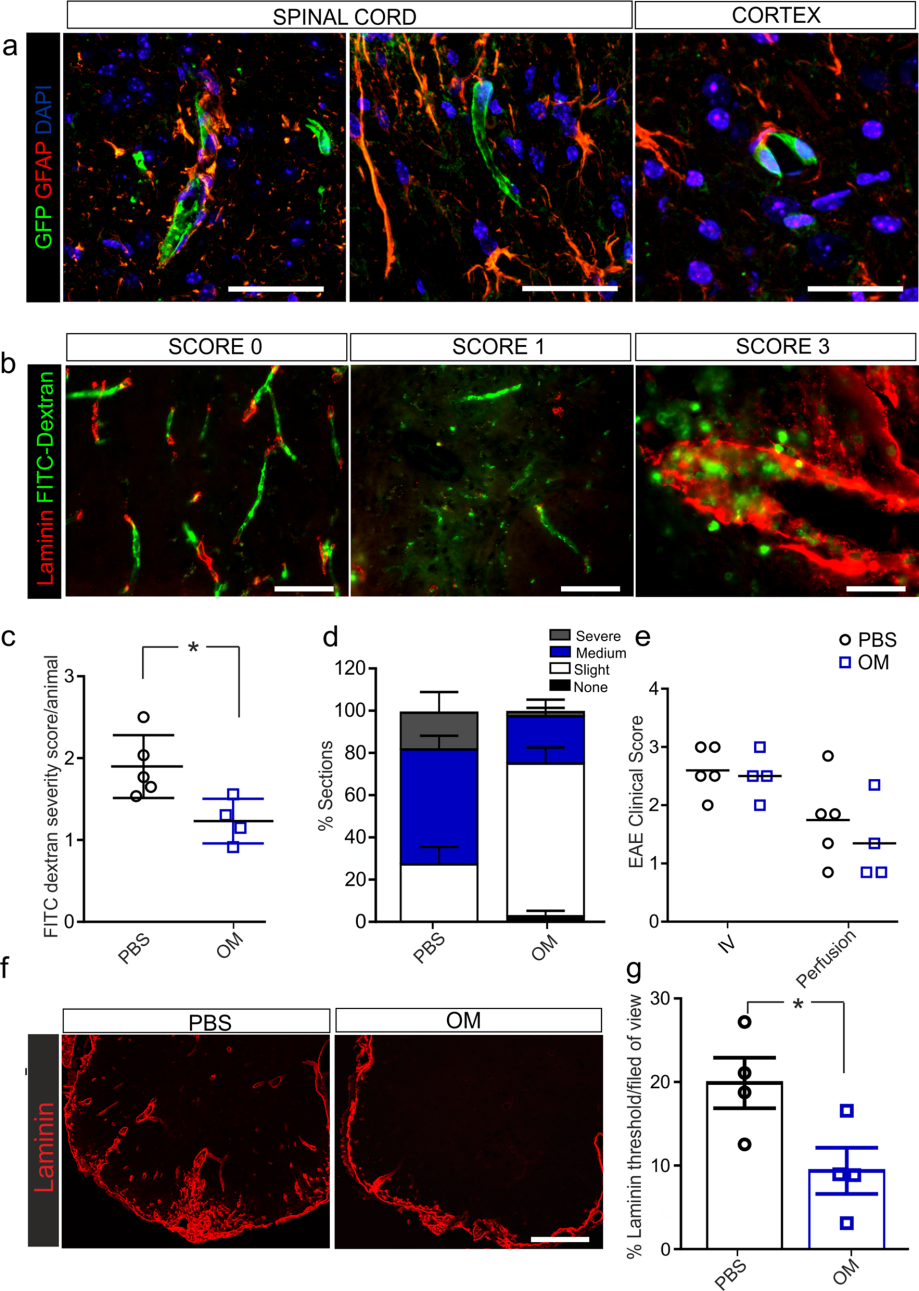
hOM-MSC localise in blood vessels and reduce blood–brain barrier (BBB) disruption within severe EAE spinal cord. **a** Immunohistochemical images of spinal cord and brain cortex of EAE mice after GFP-expressing hOM-MSCs were injected during severe disease. Only a few hOM-MSCs located in what appeared to be blood vessels lined with astrocytes (GFAP, shown in red) within spinal cord tissue and brain cortex 24 h post injection. **b** Immunohistochemical images of spinal cord after i.v. injection of FITC-Dextran dye at different levels of dextran disruption (Score 0, none; Score 1, slight; Score 3 severe) PBS, *n* = 4; hOM-MSCs, *n* = 4. **c** Average animal BBB severity score showed a significantly lower score in hOM-MSC injected animals compared to PBS controls. **d** A greater number of hOM-MSCs injected sections were scored as slight disruption compared to the PBS control animals which had a greater number of medium to severe disruption scores. **e** There were no significant differences between animal EAE clinical score at day of cell injection or at day of perfusion. **f** Laminin staining (shown in red) of severe EAE spinal cord 5 days post-cell or PBS injection. **g** Quantification of laminin expression shows hOM-MSC animals had significantly reduced levels compared to controls correlating with reduced disruption of the BBB. PBS, *n* = 4; hOM-MSCs, *n* = 4; *p < 0.05, **p < 0.01, Students unpaired t test. Scale bar represents 25 μm (**a**), 100 μm (**b**), 200 μm (**f**)

To determine whether hOM-MSCs pass through the BBB and into CNS tissue to mediate repair, hOM-MSCs were lentivirally infected to stably express GFP then injected into EAE mice during severe disease. Tissue was harvested at three-time points; 24 h, 7 days and 14 days post-transplantation. After 24 h there were only a few hOM-MSCs located in what appeared to be blood vessels lined with GFAP expressing astrocytes within spinal cord tissue and brain cortex (Fig. 3a). At 7-days or 14-days post injection there were no detectable GFP profiles within spinal cord or brain tissues. hOM-MSCs therefore appear to only localise within blood vessels and do not enter the CNS tissue or inflammatory lesions and are quickly cleared after injection.

### Analysis of blood–brain barrier (BBB) disruption with spinal cord tissue

BBB integrity was assessed using FITC-Dextran dye injection in severe EAE animals 5 days post hOM-MSC or PBS treatment before any significant changes in EAE clinical score (Fig. 3e). Sections from the entire cord of each animal were scored according to the level of dextran disruption (0, none; 1, slight; 2, medium; 3 severe, Fig. 3b). Average animal BBB severity score showed a significantly lower score in hOM-MSC injected animals compared to PBS control animals (Fig. 3c), suggesting less disruption in hOM-MSC injected animals. The percentage of sections classified into each category showed that more hOM-MSC injected animals had sections with slight disruption compared to the PBS control animals, who had a greater percentage of medium to severe disruption scores (Fig. 3d). There were also no significant differences in clinical score at day of perfusion confirming that the greater level of disruption was not a result of lower animal clinical scores (Fig. 3e). The reduction in BBB disruption 5 days post cell injection was associated with decreased laminin (Fig. 3f, g) within hOM-MSC inflammatory lesions compared to PBS control animals. This data suggests that hOM-MSCs treated animals have less disruption of the BBB than PBS-injected animals.

### hOM-MSC administration downregulates MOG-specific IL-16 cytokine response

**Fig. 4:**
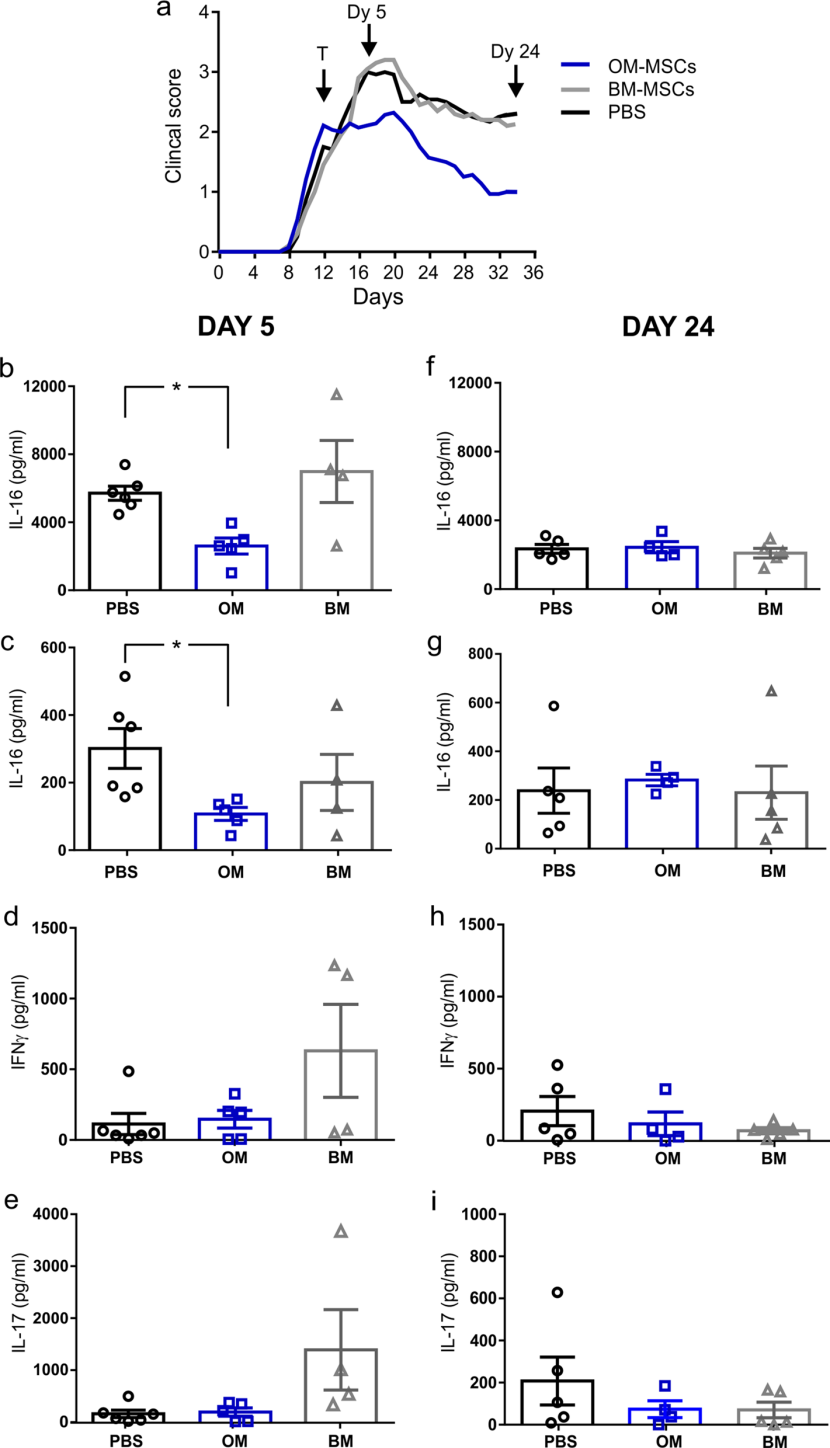
hOM-MSC treatment downregulates MOG-specific IL-16 cytokine response during severe EAE. Antigen-specific peripheral inflammatory responses were measured from lymphocytes harvested from severe EAE mice at 5 days or 24 days post hOM-MSC, hBM-MSC or PBS injection (**a** timings shown by black arrows; T: treatment, Dy 5: Day 5, Dy24: Day 24). Isolated cells were stimulated with the MOG protein (1–125) and supernatants assayed using a 35 U-plex array. IL-16 levels within serum were assayed by ELISA. hOM-MSC animals had reduced MOG-specific IL-16 production in lymphocytes (**b**) and circulating serum (**c**) compared to PBS animals at day 5 but this was not sustained until day 24 (**f**, **g**).There were no significant differences in the levels of IFNγ or IL-17 (two of the main cytokines involved in T-cell mediated disease) at day 5 (**d**, **e**, respectively) or day 24 (**h**, **i**, respectively). PBS, *n* = 6; hOM-MSCs, 5; hBM-MSCs, *n* = 4, *p < 0.05, ANOVA, Tukey’s multiple comparison

MOG specific cytokine responses in lymphocytes harvested from severe EAE animals (those treated during hindlimb paralysis) 5 days and 24 days post cell injection were investigated using the U-Plex platform (Fig. 4a). Out of the 35 cytokines analysed, only IL-16 was significantly altered in hOM-MSC treated animals compared to PBS control animals 5 days post-cell injection (Fig. 4b). Circulating IL-16 serum levels quantified by ELISA were found to also be significantly reduced in hOM-MSC injected animals compared to PBS control animals 5 days post cell injection (Fig. 4c). Interestingly, animals which received hBM-MSCs showed a trend towards increased levels of MOG-specific IFN-γ and IL-17A 5 days post cell injection compared to both PBS control and hOM-MSC animals, although not significantly different (Fig. 4d, e). However, at 24 days post-injection there were no significant differences between groups in any of the cytokines analysed (Fig. 4f–i). This data suggests that hOM-MSCs could mediate their action via suppression of IL-16 production in peripheral immune cells.

### IL-16 expression in EAE spinal cord

**Fig. 5:**
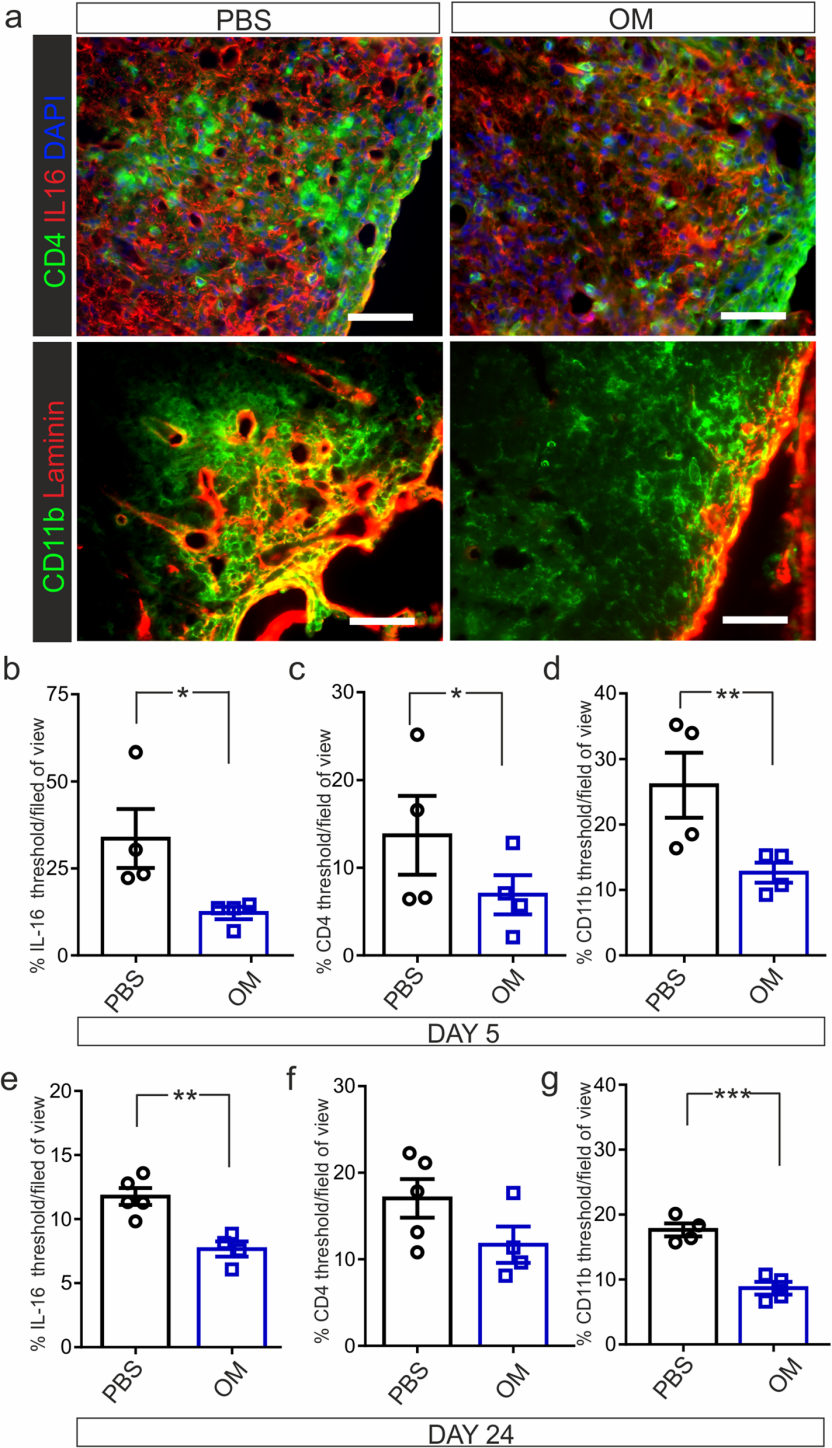
IL-16 expression in severe EAE spinal cord harvested 5- or 24-days post- cell or PBS injection. **a** Immunohistochemical images of spinal cord tissue harvested 5 days post hOM-MSC, or PBS injection stained for IL-16, CD4, CD11b and laminin. **b**–**c**. Quantification at 5 days post-hOM-MSC or PBS injection (PBS, *n* = 4; hOM-MSCs, *n* = 4). **e**–**f**. Quantification at 24 days post-hOM-MSC or PBS injection (PBS, *n* = 5; hOM-MSCs, *n* = 4). IL-16 immunofluorescence (**a**, shown in red) was significantly reduced in inflammatory lesions compared to PBS controls 5 days (**b**) and 24 days post cell injection (**e**). CD4 expression (**a** shown in green) was also reduced 5 days post cell injection (**c**), but this was not retained until 24 days (**f**). CD11b expression (**a**, shown in green) in hOM-MSC treated animals was significantly reduced at both 5 days (**d**) and 24 days (**g**) compared to PBS control. *p < 0.05, **p < 0.01, Students unpaired t test. Scale bar represents 50 μm

We next assessed whether the differences in IL-16 expression in severe EAE mice correlated with changes in CNS tissue immunohistochemistry at both 5 days and 24 days post cell injection. IL-16 immunofluorescence was significantly reduced in inflammatory lesions compared to PBS control animals at both time points, although levels were much lower at experimental endpoint (Fig. 5a, b, e). Since IL-16 is a recognised CD4 co-receptor ligand that controls the trafficking of CD4 T-cells, we also assessed CD4 expression in inflammatory lesions. There were reduced levels of CD4 at the early time point in hOM-MSC injected animals compared to PBS control (Fig. 5a, c), however this was not detected at day 24 (Fig. 5f). CD11b, a leukocyte and microglial marker, was also significantly decreased in hOM-MSC injected animals compared to PBS control animals at both time points analysed (Fig. 5a, d, g). This suggests that the lower levels of inflammatory cells found within hOM-MSC injected animals (shown in Fig. 2d-f) reflects a reduction of mature myeloid cells which are known to be a prominent component of EAE inflammatory infiltrates.

### In vitro investigation of IL-16 during demyelination

**Fig. 6:**
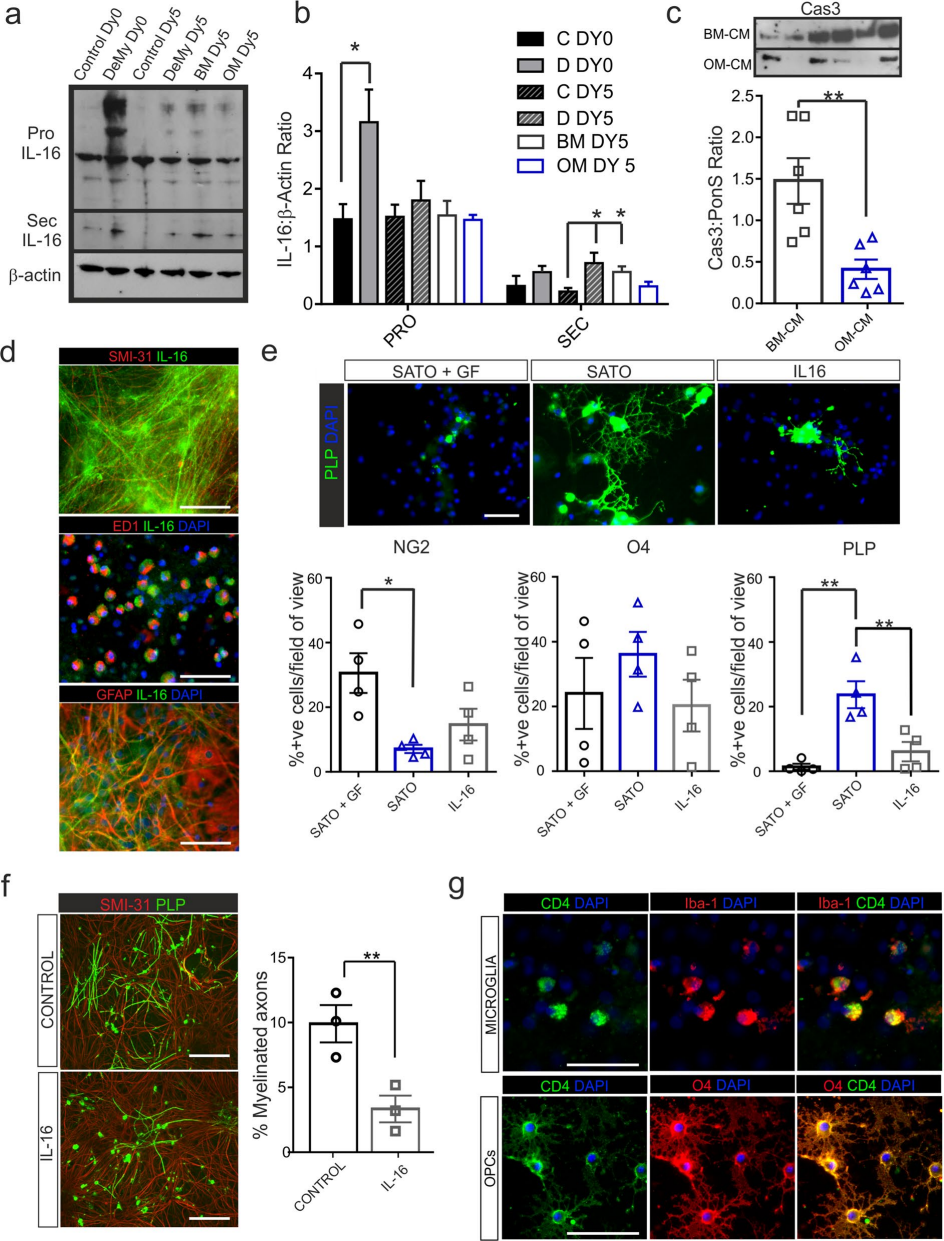
IL-16 expression is upregulated during in vitro demyelination and inhibits OPC differentiation and myelination. **a** Representative Western blot image of IL-16 expression in CNS control myelinating cultures (Control Dy 0) and 24 h after demyelination with anti-MOG and complement (DeMy 0). After DeMy, cultures were treated for 5 days with hOM-MSC-CM (OM Dy5) or hBM-MSC-CM (BM Dy5) or treated with media alone (Control Dy5). **b** Western blot quantification revealed the predominant form of IL-16 expressed by the cultures was Pro IL-16, which was significantly upregulated after DeMy. hOM- or hBM-MSC-CM treatment for 5 days post DeMy had no effect on Pro IL-16 expression. The bioactive secreted form of IL-16 (Sec IL-16) was expressed at very low levels in control cultures (Control Dy 0, C Dy 0) but was significantly upregulated after demyelination (DeMy Dy5, D Dy5). hOM-MSC-CM treated cultures (OM Dy5) expressed low levels however, those treated with hBM-MSC-CM (BM Dy5) maintained the higher expression of IL-16 found in DeMy cultures (D Dy5). *n* = 5, *p < 0.05, **p < 0.01, ANOVA, Tukey’s multiple comparison. **c** Western blot analysis of Caspase-3 (Cas3) expression in hOM- and hBM-MSC-CM. hOM-MSCs secreted lower amounts compared to hBM-MSCs. Secreted levels were standardised to Ponceau S (PonS) staining of the membrane prior to staining. hOM-MSC-CM, *n* = 6; hBM-MSC-CM, *n* = 6 **p < 0.01, Student’s unpaired t test. **d** IL-16 was expressed in axons and microglia, however astrocytes produced only negligible amounts. **e** Immunohistochemical images of purified OPCs grown in growth factors (GF) that retain their progenitor state (SATO + GF) or grown in media that allows differentiation (SATO) or after treatment with IL-16 (100 ng/ml in SATO). Proteolipid protein (PLP), **a** late OPC marker, is shown in green and cell nuclei are stained by DAPI in blue. **b** Quantification of the percentage of positive OPCs stained for NG2 (early marker), O4 (middle maker) or PLP (late marker). NG2 expression was significantly reduced and PLP was significantly increased in SATO compared to SATO + GF correlating with enhanced OPCs differentiation. IL-16 treatment had no effect on NG2 or O4 expression but caused a significant reduction in the number of mature PLP positive OPCs compared to SATO. There were no significant differences in OPC numbers between treatments (*n* = 4, all conditions). **f** Immunohistochemical images of control CNS myelinating cultures or after IL-16 (100 ng/ml) treatment from Day 12. Axons are stained with SMI-31 (shown in red) and myelin stained with PLP (shown in green). Quantification revealed that IL-16 treatment from day 12, significantly reduced the number of myelinated axons compared to control cultures (*n* = 3, all conditions). **g** Immunohistochemical images of CD4 expression (shown in green) in mircoglia and OPCs. CD4 was upregulated in microglia after demyelination (Iba-1 shown in red). Purified OPCs (O4 shown in red) expressed CD4 in a peri-nuclear localisation. Scale bars represents 50 μm in all images

To examine the role of IL-16 in vitro, CNS spinal cord cultures were demyelinated (DeMy) then treated with hOM- or hBM-MSC-CM and IL-16 protein levels examined via Western blot (Fig. 6a). We found that immediately after demyelination there was a significant increase in Pro IL-16 in control cultures (Fig. 6a, b). The lower molecular weight, bioactive secreted form of IL-16 was also significantly upregulated 5 days after demyelination compared to non-demyelinated control cultures (Fig. 6a, b). hOM-MSC treated cultures had lower levels of bioactive IL-16 similar to non-demyelinated controls, whilst those treated with hBM-MSC-CM retained significant upregulation of bio-active IL-16 comparable to demyelinated control cultures. This suggests that there is less bioactive IL-16 available after demyelination during hOM-MSC treatment. Under resting conditions, IL-16 was detected diffusely along axons but predominantly in microglia; astrocytes had very little IL-16 staining (Fig. 6d). Immediately after demyelination IL-16 staining was upregulated in ameboid shaped microglia, suggesting that microglia are the predominant producers of IL-16 immediately after injury (Fig. 6d). Activation of pro-IL-16 occurs by caspase-3 dependent cleavage, therefore levels of caspase-3 secretion was investigated in hOM- and hBM-MSC-CM. It was found that hBM-MSC-CM had much higher expression of caspase-3 than hOM-MSC-CM (Fig. 6c).

### Effect of IL-16 on OPC differentiation and myelination

Since IL-16 upregulation occurred during demyelination in vitro, we examined whether it could impact OPC differentiation and/or de novo myelination. The exogenous addition of IL-16 to purified OPCs caused a significant reduction in the number of mature PLP stained OPCs compared to control cultures (Fig. 6e). Although there was a trend towards less OPCs after IL-16 treatment, this was not significantly different. In addition, exogenous addition of IL-16 to CNS cultures during the period when developmental myelination occurs, revealed a reduction in the number of myelinated axons compared to control cultures (Fig. 6f). Bioactive IL-16 binds to the CD4 receptor to cause subsequent signalling, therefore CD4 expression was also assessed. As expected, microglia upregulated CD4 after demyelination, however purified OPCs also were found to express CD4 in a peri-nuclear localisation (Fig. 6g). This data suggests a novel role for IL-16 in negatively controlling oligodendrocyte differentiation and myelin sheath formation.

## Discussion

Human trials of autologous transplantation of MSCs in secondary progressive MS patients has illustrated their safety, strongly supporting their future use as treatment [10, 12, 13]. Previously, we reported the therapeutic benefit of MSCs from biopsies of human olfactory mucosa for CNS repair which have similar biological and antigenic characteristics as hBM-MSCs, but also promote myelination in vitro [14, 15] and in vivo [22]. In this investigation, we compared the ability of hBM-MSCs and hOM-MSCs to ameliorate EAE, an animal model of MS. We initially tested whether either cell type would influence disease outcome when administered during mild disease (loss of tail tone). hOM-MSCs and hBM-MSCs both ameliorated disease severity when injected shortly after disease onset, but only hOM-MSCs were effective once animals had developed severe disease (hind limb paralysis). hOM-MSC enhanced recovery was associated with significant reductions in inflammatory cell infiltration into spinal cord lesions, specifically reduced numbers of CD45 lymphocytes and CD11b macrophages/microglia and dendritic cells which are known to be prominent components of the EAE inflammatory infiltrate [34]. Furthermore, animals had less axonal pathology compared to either PBS or hBM-MSC transplanted animals correlating with improved clinical scores.

Many different types of human adult-tissue-derived MSCs have been shown to have therapeutic potential in the EAE model, although there has been large variability in the efficacy between cell types [7, 35–39]. Tissue-specific stem cells support the tissue from which they originate, suggesting that certain MSC types might be more suitable for the treatment of EAE [40]. hOM-MSCs reside in a neurogenic niche vulnerable to physical and chemical injury that can undergo continuous cell replacement after injury. hOM-MSCs are widely distributed throughout the highly accessible olfactory mucosa [41] and can be grown in large numbers owing to their fast proliferation rate, almost 8 times faster than hBM-MSCs [14]. The entire hOM-MSC population highly express nestin, while hBM-MSCs express substantially less (approximately 50%) [42]; typically expressed in those hBM-MSCs associated with adrenergic nerve fibres [43]. This may reflect functional differences since nestin + MSCs are considered to have a neurosupportive role, making them more suitable for MS repair [21, 40]. hOM-MSCs have also been shown to secrete lower levels of the inflammatory cytokines IL-6, IL-8 and CCL2 constitutively when compared to hBM-MSCs and their CM can skew microglia to an anti-inflammatory phenotype [15]. In addition, hOM-MSCs suppress the cytotoxic function of CD8 + lymphocytes and natural killer cells, illustrating immunomodulatory function [44, 45].

Although there are numerous reports of the beneficial effect of BM-MSCs in EAE animals, this has often been evident when injected before or at the onset of disease [7, 46] and/or via an intraperitoneal route [8, 47]. In fact, the importance of timing during BM-MSC i.v. administration has been reported to be crucial. When injected at the peak of disease or at the time of EAE stabilisation, BM-MSCs were inefficient in reducing the clinical score and induced “atypical” symptoms such as unbalanced gait or rotatory defects, but not if administered during early time points [48]. This was thought to be due to the predominance of Th17 lymphocytes [49], contrary to classic EAE, which is governed by Th1 lymphocytes [48]. Furthermore, reports have shown that BM-MSCs can exacerbate EAE by increasing T-cell brain infiltration [50–54]. In this investigation, we have also shown that hBM-MSCs are only efficacious when given at early onset of disease and although we did not see exacerbation of EAE score compared to control animals, there was a trend towards increased levels of pro-inflammatory cytokines in hBM-MSC transplanted animals during severe disease. This data suggests that the inflammatory status is an important consideration for administering hBM-MSCs as a therapeutic strategy.

The EAE model is predominantly due to inflammation caused by actively induced autoreactive T-cells, that firstly collect in the spleen [55, 56]. They migrate into the CNS and recognise their cognate antigen on local antigen presenting cells (APCs) activating an inflammatory cascade leading to tissue injury. CNS tissue debris can be found in APCs in the cervical and lumbar lymph nodes and the spleen [57]. Further T-cell responses are triggered in these tissues leading to new autoreactive T-cell specificities that exacerbate the ongoing autoimmune reaction [57, 58]. Since hOM-MSCs can ameliorate severe disease when there is already active tissue damage, it is possible that they modulate further T-cell responses trigged within either the lymph nodes or spleen, reducing the number of new T-cells generated. Indeed, biodistribution studies have shown that although MSCs accumulate firstly in the lungs within a few hours after i.v. infusion [59–61] they relocalise predominantly in the spleen [39, 62]. It is also considered that the number of recirculating MSCs remain low [63] and that infused cells can undergo apoptosis [64, 65]. This could trigger a response in macrophages who adapt their immunoregulatory function after the phagocytosis of dead MSCs [65, 66]. We found little evidence of GFP tagged hOM-MSCs present after 24 h, suggesting they do not survive long term in recipient animals. Although secondary homing to inflammatory or injured sites has been shown to occur, likely due to BBB disruption [1, 2, 4, 39, 67], in this investigation we found no hOM-MSCs within brain or spinal cord sections; only a few GFP profiles were detected within what appeared to be blood vessels. hBM-MSCs and human embryonic derived MSCs (hES-MSCs) have been shown to home to the CNS microvasculature, however only hES-MSCs had the capacity to extravasate and migrate into the parenchyma [54].

A potential mechanism owing to hOM-MSC therapeutic action may be directly at the BBB, since animals had less laminin and FITC dextran disruption compared to control animals at early time points. During EAE, leukocyte recruitment occurs exclusively around endothelial cell basement membranes which contain laminin, a major functional component of all basement membranes [68]. In this investigation, there was extensive upregulation of laminin in PBS animals, as shown previously in acute and particularly, chronic EAE lesions [26, 69]. Higher densities of inflammatory cells were associated with increased laminin deposition, as shown during the peak phase of EAE previously [26]. Still, hOM-MSC treated animals had significantly less laminin expression which correlated with less dextran disruption and reduced inflammatory infiltration, illustrating their therapeutic role either directly on the recruitment of leukocytes across the BBB and/or directly modulating BBB permeability. In models of brain inflammation or haemorrhage MSCs have also been shown to stabilise the BBB through their regulation of astrocyte reactivity, leading to reductions in neutrophil infiltration [70, 71]. It is therefore tempting to speculate that hOM-MSCs mediate their action directly by modulation of BBB permeability, perhaps mediating astrocyte reactivity in a similar manner, but this requires further investigation. hOM-MSCs may therefore exhibit a cell autonomous effect within the periphery and not directly mediate their action due to engraftment within the CNS compartment.

### Involvement of IL-16 in hOM-MSC mechanism-of-action

The investigation of antigen-specific peripheral inflammatory responses revealed IL-16 as the only cytokine differentially downregulated in hOM-MSC transplanted animals versus significantly increased in PBS and hBM-MSC transplanted animals. Further investigation confirmed reduced IL-16 expression within serum and spinal cord tissue 5 days post hOM-MSC transplantation. IL-16 has already been implicated in both EAE and MS pathology. In EAE experiments in which the activity of IL-16 was blocked using a neutralisation antibody, there were reductions in CD4 + T cell infiltration, less demyelination and axonal loss [72]. Furthermore, animals treated with anti-IL16 showed efficient amelioration of relapsing disease demonstrating the significance of this cytokine in EAE [72]. Similar to our own investigation, EAE mice spinal cords contained high levels of IL-16 which correlated with disease severity [72]. Interestingly, in MS patient brain and spinal cords, IL-16 expression correlated with CD4 + Th1 inflammation and phosphorylation of axonal cytoskeleton in inflammatory lesions [73] with immunoreactivity confined to infiltrating mononuclear cells [73]. Recently, single-nucleotide polymorphisms in the IL-16 gene have also correlated with increased serum levels of IL-16 in MS patients however, further studies in different populations are needed to establish this as a marker for genetic susceptibility [74]. Collectively, this data suggests an important role for IL-16 signalling in EAE and MS.

IL-16 is a proinflammatory cytokine generated by caspase-3-dependent cleavage of pro-IL-16. It therefore exists in different molecular weight forms; a large molecular weight precursor form (pro-IL16, 80 kDa) that after activation forms intermediate products and a bioactive form (secreted IL-16, 17 kDa). Bioactive IL-16 then binds to the CD4 receptor and causes subsequent signalling [75]. Interestingly, it is expressed in both the immune and nervous systems. Typically produced by T lymphocytes, monocytes/macrophages, dendritic cells, mast cells, fibroblasts and microglia [72, 76], a larger splice variant known as neuronal IL-16 (NIL-16) has been found within CD4 + granule neurons in the cerebellum and hippocampus [77]. In EAE lesions, IL-16 is considered to be produced predominantly by the CD45 + infiltrating immune cells and the resident CD11b + microglia, the main cell populations to be modulated by hOM-MSC transplantation [78]. Once secreted, bioactive IL-16 has diverse immune-regulatory functions including chemotaxis of CD4 + T cells, monocytes, and eosinophils, expansion of memory effector T cells, and activation of antigen-presenting cell functions [79]. We found that in CNS myelinating cultures, both before and after demyelination, the predominant cell type to produce IL-16 was microglia. After demyelination, there was significant upregulation of pro IL-16 within inflammatory microglia, however the levels of bioactive IL-16 only significantly increased 5 days later. hOM-MSC-CM treatment of demyelinating cultures contained similar levels of pro IL-16, although produced less bioactive IL-16 suggesting a direct effect on IL-16 cleavage and availability. Since IL-16 is generated by caspase-3-dependent mechanisms it was interesting to find that hOM-MSCs secreted significantly less caspase-3 than hBM-MSCs which could correlate to the reduced available levels in demyelinating cultures. However, the direct action on IL-16 bioavailability could be entirely independent of the effect elicited in vivo, where hOM-MSCs likely mediate their effect in the peripheral compartment.

Further in vitro investigation of IL-16 revealed a direct negative impact on the number of mature PLP expressing oligodendrocytes and reduced levels of de novo myelination. To our knowledge, this is the first investigation to reveal an inhibitory role for IL-16 on myelinating glia, however it is well established that chemokine receptors are present not only on inflammatory cells, but also on astrocytes, oligodendrocytes, and neurons [80]. The receptor for IL16 is CD4 and although typically its expression is found on immune cells it has been reported in neurons, glia, and microglia throughout the brain [81–84]. In this study, we found CD4 expression predominantly in microglia within CNS cultures, however there was diffuse peri nuclear CD4 expression on purified OPCs. This supports the hypothesis that the IL-16/CD4 axis is important in the control of myelination, although whether directly via CD4 receptors on OPCs or indirectly via other cell types such as microglia or astrocytes requires further investigation.

This investigation has shown that hOM-MSC and not hBM-MSC treatment, ameliorates EAE when administered during severe disease, likely through immunomodulation of cells that produce IL-16. IL-16, which is upregulated in EAE lesions and after demyelination in vitro, has a direct detrimental effect on oligodendrocyte differentiation and myelination. Therefore, hOM-MSCs may be beneficial for the treatment of MS.

## Data Availability

Raw data images and files analysed during the current study are available from the corresponding author on reasonable request.
